# Gains in the current understanding of managing neovascular AMD with brolucizumab

**DOI:** 10.1186/s12348-023-00369-8

**Published:** 2023-11-23

**Authors:** Bahram Bodaghi, Arshad M. Khanani, Ramin Khoramnia, Carlos Pavesio, Quan Dong Nguyen

**Affiliations:** 1 Department of Ophthalmology & Visual Sciences, Sorbonne University, Paris, France; 2 Sierra Eye Associates, Reno, NV, USA; 3 University of Nevada, Reno School of Medicine, Reno, NV USA; 4 Department of Ophthalmology, Heidelberg University, Heidelberg, Germany; 5 Department of Uveitis, Moorfields Eye Hospital and University College London, London, UK; 6 Byers Eye Institute, Stanford University School of Medicine, 2370 Watson Court, Suite 200, Palo Alto, CA 94303 USA

## Abstract

**Background:**

Unresolved retinal fluid and high injection burden are major challenges for patients with neovascular age-related macular degeneration. Brolucizumab addresses these challenges by providing robust vision gains and superior fluid resolution, with the potential for longer treatment intervals.

Brolucizumab has been associated with adverse events of retinal vasculitis and retinal vascular occlusion typically in the presence of intraocular inflammation (IOI). To define the incidence of the adverse events, Novartis convened an external safety review committee, which found a rate of 4.6% for definite or probable IOI, 3.3% for retinal vasculitis, and 2.1% for retinal vascular occlusion in the HAWK and HARRIER trials. Novartis also established a coalition to explore 4 areas regarding the adverse events: root cause, patient characterization, event mitigation and vigilance, and treatment protocols for the adverse events. Based on the coalition findings, a risk mitigation framework was developed. Prior to initiating treatment with brolucizumab, it is important to weigh the potential benefit against risk of adverse events and to consider patient risk factors such as prior history of IOI and/or retinal vascular occlusion. To mitigate the potential for IOI-related adverse events, it is important to conduct a thorough dilated eye examination before each injection and closely monitor patients throughout treatment. Patients should be educated on symptoms of IOI to monitor for. Brolucizumab should not be injected in the presence of active IOI. If an adverse event is identified, prompt and intensive treatment should be considered.

**Conclusion:**

Progress has been made in understanding how to mitigate IOI-related adverse events following treatment with brolucizumab.

Despite the benefit of anti–vascular endothelial growth factor (anti-VEGF) therapy, significant unmet needs exist in the management of patients with neovascular age-related macular degeneration (nAMD). Patients with nAMD may have unresolved fluid, even with monthly injections [1]. In a retrospective analysis of electronic health records from the US Retina database, more than 50% of eyes were found to have residual retinal fluid after 2 years [2]. High injection burden and treatment adherence are also major challenges for patients [3–5]. An analysis of real-world data from the IRIS Registry showed that, at the end of year 1, almost 40% of patients were receiving intravitreal injections more frequently than every 8 weeks [4]. A systematic review identified nonadherence as a prevalent problem, with up to 57% nonadherence at 1 year [5]. Undertreatment due to nonadherence may lead to long-term vision loss [6].

In 2019, brolucizumab received US Food and Drug Administration (FDA) approval for treatment of nAMD, and was subsequently approved in more than 40 countries [7]. Brolucizumab offers an important treatment option for patients with nAMD. The HAWK and HARRIER trials demonstrated that brolucizumab (q8 or q12 weeks) was noninferior to aflibercept (q8 weeks) in visual acuity gains at week 48 [8], and these gains were maintained out to week 96 [9]. Brolucizumab also showed greater reduction in central subfield thickness than was seen with aflibercept [8, 9], and rates of intraretinal fluid and subretinal fluid presence were lower in brolucizumab-treated eyes than in aflibercept-treated eyes [8, 9]. Furthermore, approximately half of patients who were treated with brolucizumab were maintained on 12-week dosing intervals after the initial loading dose, through week 48 [8].

Brolucizumab demonstrated an overall favorable benefit-risk profile in the HAWK and HARRIER trials. At 96 weeks, pooled data showed intraocular inflammation (IOI) in 4.5% of eyes and retinal artery occlusion in 0.9% of eyes treated with brolucizumab 3 mg or 6 mg compared with 0.8% and 0.1%, respectively, of eyes treated with aflibercept 2 mg [9, 10]. Despite these events, visual acuity outcomes were comparable between brolucizumab and aflibercept in both trials [9].

After the FDA approval of brolucizumab [11], postmarketing reports led to an emerging safety signal for adverse events (AEs) of retinal vasculitis and retinal occlusive vasculitis [12–14]. To further define the incidence of these AEs and the risk of vision loss, Novartis convened an external safety review committee (SRC) composed of global retina and uveitis specialists, imaging experts, and ophthalmology experts from 2 separate external data monitoring committees [15]. The SRC conducted an independent unmasked post hoc review of the investigator-reported cases of IOI, retinal artery occlusion, and endophthalmitis from the HAWK and HARRIER trials [12, 15]. The SRC reviewed patient images and determined whether cases were likely to be drug related and within the spectrum of IOI, retinal vasculitis, and/or retinal vascular occlusion, regardless of the *Medical Dictionary for Regulatory Activities* (MedDRA) terminology used in the trials. The SRC found a rate of definite or probable IOI of 4.6% in the HAWK and HARRIER trials, which was similar to the incidence of IOI (4.5%) reported by the study investigators [12, 15]; a rate of retinal vasculitis of 3.3%; and a rate of retinal vascular occlusion of 2.1%, which was higher than that reported by the investigators [12, 15]. The rate of retinal vasculitis and retinal vascular occlusion may have been higher for the SRC because it was conducting an extensive and thorough review of the cases, with definitions of the events and outcomes proposed a priori and evolving during the review based on the observations made by the SRC [15]. The incidence of at least moderate vision loss associated with IOI was < 1% in each of the brolucizumab and aflibercept groups. In addition, the overall incidence of moderate or severe vision loss (including that associated with definite or probable IOI, retinal vasculitis, and/or retinal occlusion) was similar for brolucizumab- and aflibercept-treated eyes (7.4% and 7.7%, respectively) [15].

In addition to the SRC, Novartis established a coalition composed of a fully dedicated internal team of 150 Novartis associates, who worked with more than 40 external medical experts from leading universities, hospitals, medical centers, and clinics around the world to explore 4 key areas regarding the AEs: the root cause of the AEs, patient characterization, event mitigation and vigilance, and treatment protocols for the AEs [10, 12]. Findings from the coalition workstreams have contributed to a better understanding of the AEs and helped provide physicians with the information they need to make informed treatment decisions at each step of the patient journey. Based on the coalition findings, a risk mitigation framework was developed.

## Mitigation framework for adverse events following brolucizumab injection

### Patient selection

Patients who have persistent retinal fluid and are showing deterioration in vision because of uncontrolled disease with other therapies should be considered as possible candidates for treatment with brolucizumab. Before treatment is initiated, it is important to weigh the potential benefits of brolucizumab against the risks of retinal vasculitis, retinal vascular occlusion, and vision loss. Prior history of IOI, retinal vasculitis, and/or retinal vascular occlusion in the previous 12 months has been identified as an important potential risk factor for IOI-related AEs following brolucizumab [16, 17]. Female sex has also been identified as a weaker potential risk factor [16, 17]. Patients with active IOI, retinal vasculitis, and/or retinal vascular occlusion should not be injected with brolucizumab [11, 18]. Physicians should discuss with the patient the potential benefits and risks of brolucizumab, so the patient understands the potential benefit of better disease control and, at the same time, the risk of retinal vascular occlusion and vision loss with brolucizumab.

### Event mitigation and vigilance

A thorough dilated eye examination should be conducted before each brolucizumab injection, and patients should be closely monitored throughout treatment [19–21]. The examination should include visualization of the anterior chamber, vitreous, and retina. Patients with active IOI, retinal vasculitis, and/or retinal vascular occlusion should not be treated with brolucizumab [11, 18]. In the HAWK and HARRIER studies, approximately three quarters of the IOI-related AEs occurred in the first 6 months of treatment [15]. Patients should be educated on symptoms to monitor, including changes in visual acuity, eye pain, floaters, discomfort, or ocular hyperemia, to help with early identification of any AEs [11, 20].

### Treatment of the adverse events of interest

In the event of IOI, retinal vasculitis, or retinal vascular occlusion, prompt and intensive treatment should be considered, [7, 19] applying standard-of-care guidelines. Intensive treatment may include multimodal topical, systemic, and intravitreal steroids, depending on the presentation of the inflammation [7]. Treatment with brolucizumab should be discontinued following IOI, including retinal vasculitis and/or retinal vascular occlusion [18]. In a post hoc analysis of HAWK and HARRIER, Singer et al. found that most events of IOI were managed conservatively, and recommended vigilance and prompt treatment [21]. Real-world evidence from independent publications has shown that it is possible for IOI-related AEs following brolucizumab to be managed with intensive treatment, with reversal of reduced visual acuity possible [22–25].

## Potential mechanistic drivers of adverse events following brolucizumab injection

A thorough root-cause analysis was performed to identify, characterize, and prioritize potential mechanistic drivers of the AEs of interest following treatment with brolucizumab. The parameters studied included but were not limited to manufacturing, pharmacology, antidrug antibodies, neutralizing antibodies, and other immune-mediated mechanisms [17]. Immunogenicity occurs when there is an immune response against a therapeutic protein, leading to production of antidrug antibodies [26]. Consequences of immunogenicity can include lack of evidence of clinical effect, loss of efficacy, or serious AEs [26]. Findings have shown that immunogenicity against brolucizumab appears to be necessary for developing retinal vasculitis and/or retinal vascular occlusion with brolucizumab. However, unknown factors must also play a role, given that many patients with antidrug antibodies do not develop retinal vasculitis or retinal vascular occlusion following treatment with brolucizumab [27, 28].

## Case study 1: brolucizumab used to increase nAMD treatment durability

**Fig. 1:**
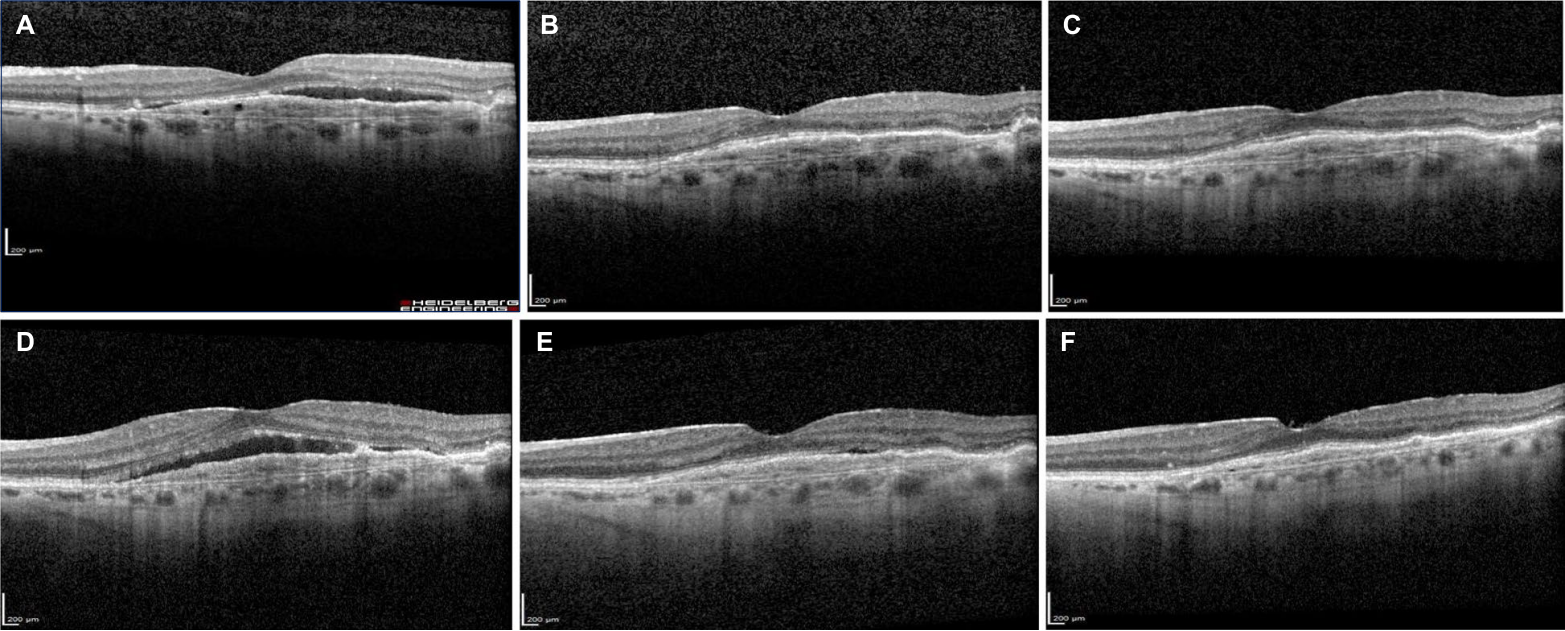
OCT B-scans of a patient switched to brolucizumab to increase the treatment interval duration. **A** On August 5, 2019, 6 weeks after an aflibercept injection, subretinal fluid was apparent. **B** On October 16, 2019, 5 weeks after the next aflibercept injection, disease activity was controlled. **C** On January 27, 2020, 14 weeks after the first brolucizumab injection, the patient showed good disease control. **D** On May 19, 2020, 15 weeks after the second brolucizumab injection—a longer treatment interval than recommended—disease activity had returned. **E** On August 28, 2020, 13 weeks after the third brolucizumab injection, there was trace subretinal fluid. **F** On November 6, 2020, 10 weeks after the fourth brolucizumab injection, there was no disease activity. OCT, optical coherence tomography

The following case study provides an example of the use of brolucizumab to increase treatment durability. A 70-year-old woman, who was diagnosed with nAMD in 2013, required intravitreal injections every 4 to 5 weeks. Treatment history included 15 injections of bevacizumab, 8 injections of ranibizumab, and 37 injections of aflibercept. She received 8 injections of aflibercept in 2019. On August 5, 2019, 6 weeks after an aflibercept injection, the patient showed disease activity, with subretinal fluid on optical coherence tomography (OCT) and best corrected visual acuity (BCVA) of 20/30 (Fig. 1A). Because there was disease activity, the treatment interval was decreased to 5 weeks. On October 16, 2019, 5 weeks after the aflibercept injection, disease activity was deemed well controlled per OCT, with BCVA of 20/30 (Fig. 1B); however, there was a question of whether the patient wanted to try brolucizumab as a more durable treatment option. At the time, the community lacked awareness of the AEs of retinal vasculitis and retinal vascular occlusion with brolucizumab. At that appointment, the patient decided to start treatment with brolucizumab and was monitored every month until 8 weeks and then monitored every 2 weeks. The patient reached 14 weeks (January 27, 2020) with good disease activity control, no fluid, and stable visual acuity following a single brolucizumab injection (Fig. 1C), thereby more than doubling the treatment interval with brolucizumab compared with aflibercept. Once the risk of retinal vasculitis and retinal vascular occlusion became known, the patient was informed of the risk and asked if she wanted to continue with brolucizumab. The patient chose to continue because of the durability benefits; she was then educated on the signs and symptoms of IOI to monitor for (changes in visual acuity, eye pain, floaters, discomfort, and ocular hyperemia). Following the second injection of brolucizumab, the patient returned to the clinic after 15 weeks (May 19, 2020), which was a longer interval than recommended, and had recurrent disease activity, with decreased BCVA of 20/50 (Fig. 1D). The patient decided to continue brolucizumab, and the clinic ensured that she returned in a timely fashion. Thirteen weeks after the third brolucizumab injection (August 28, 2020), there was trace subretinal fluid on OCT, BCVA of 20/40^+1^ (Fig. 1E). Ten weeks after the fourth injection (November 6, 2020), the patient had no disease activity and BCVA of 20/40^−1^ (Fig. 1F). The patient continued to have good disease control and good visual acuity with brolucizumab administered every 10 to 12 weeks (January 22, 2021, BCVA: 20/30^−2^; April 2, 2021, BCVA 20/30^+1^). Every time this patient comes to the clinic, the eye is dilated and examined for inflammation so that any AEs can be promptly treated. A discussion about the risks and benefits of brolucizumab occurs at every visit. The patient reports benefit from coming to the clinic every 3 months instead of every 4 to 5 weeks.

## Case study 2: treatment of retinal vasculitis following brolucizumab injection

**Fig. 2:**
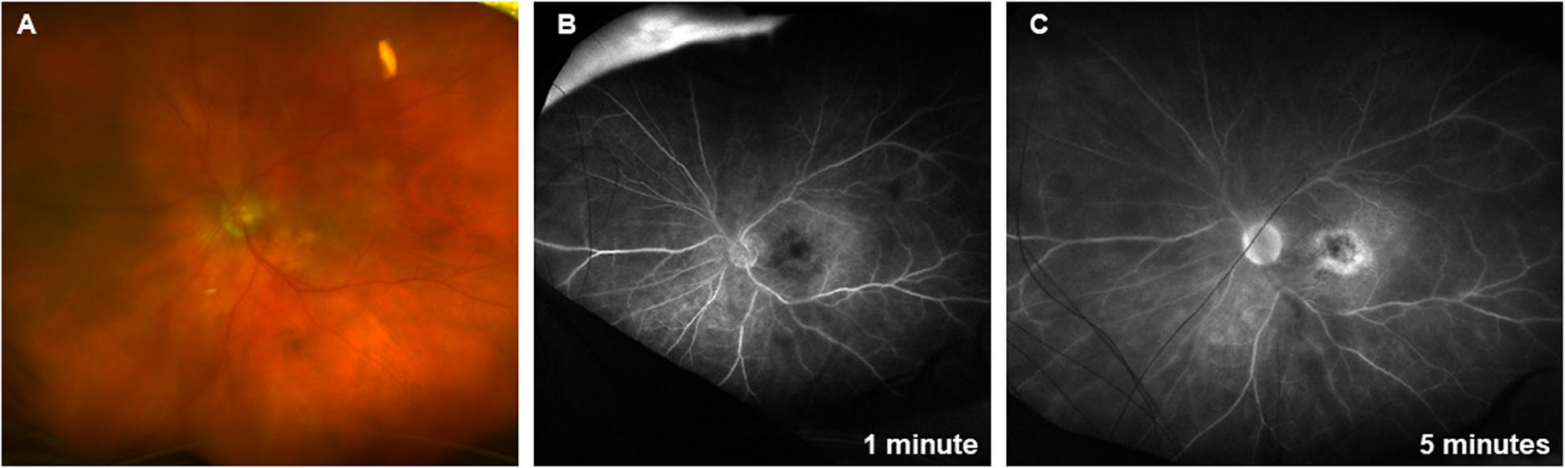
Panuveitis with nonocclusive vasculitis in the left eye following injection with brolucizumab. **A** Fundus examination showed vitreous haze, hyperemia of the optic nerve, and sheathing around some of the retinal vessels. **B**, **C** Fluorescein angiography showed optic nerve leakage and perivascular leakage in the posterior pole and peripheral retina

The following case study describes retinal vasculitis and its treatment following an injection of brolucizumab. An 88-year-old Caucasian woman presented to an urgent care eye clinic on October 6, 2021, with sudden onset of severe pain and decreased vision 3 weeks after the first intravitreal injection of brolucizumab OS. The patient had a history of nAMD OS and non-nAMD OD. The patient’s treatment history included photodynamic therapy and repeated intravitreal injections of ranibizumab and aflibercept OS; however, the nAMD was not under control. Brolucizumab was employed with the goal of achieving superior control of disease activity. Three weeks before presentation, BCVA was 20/50 OD and 20/500 OS. In both eyes, the cornea and anterior vitreous were clear, the anterior chamber was deep and quiet, and the intraocular pressure was normal (11 mm Hg). Three weeks after injection with brolucizumab OS (at the time of the urgent visit to the clinic), BCVA OS dropped to counting fingers. Keratic precipitates were visible in the cornea. The anterior chamber showed 3 + cells and 2 + flare and the anterior vitreous showed 3 + cells and 3 + haze. Intraocular pressure remained at 11 mm Hg. Fundus examination showed vitreous haze, optic nerve hyperemia, and retinal vessel sheathing (Fig. 2A). Fluorescein angiography showed optic nerve leakage and perivascular leakage of the vessels in the posterior pole, as well as in the peripheral retina (Fig. 2B, C). OCT examination showed the foveal contour was semi-preserved, but there was subretinal fluid. No inflammatory findings or vascular leakage were found in the right eye. Based on the clinical findings and the timing of the AEs, the patient was diagnosed with brolucizumab-induced panuveitis with nonocclusive vasculitis in the left eye. Given her age and social circumstances, the patient was admitted to the hospital, and treatment with intravenous methylprednisolone infusions (750 mg per day for 3 days) was initiated. The decision to use systemic rather than intravitreal steroids at the acute stage was made to avoid worsening of the disease in case the etiology was infectious. In addition, because of the anterior segment inflammation, the patient was started on prednisolone acetate qid. Timolol bid was employed to protect the pressure; a dilating drop (atropine bid) was also employed. One day after the first methylprednisolone infusion, the patient reported symptomatic improvement, with no pain and improved blurriness; however, ocular examination findings remained the same. Three days after the first infusion, visual acuity remained at counting fingers, but keratic precipitates, anterior chamber cells and flare (1 + cells, 1 + flare), and anterior vitreous cells and haze (2 + cells, 2 + haze) had improved. The patient noted visual improvement. However, she developed a psychotic AE (thoughts of jumping from the window), which was thought to be secondary to the steroids. Therefore, even though the methylprednisolone infusions were stopped, the patient was not started on oral systemic steroid therapy; instead, an intravitreal dexamethasone implant was provided. At that time, the inflammation was thought not to be of infectious etiology. Ten days after the dexamethasone implant, visual acuity remained at counting fingers, the corneal keratic precipitates had decreased, the anterior chamber was deep and quiet, and the anterior vitreous had improved to 0.5 + cells and 1 + haze. Although vision did not recover, aggressive treatment was nevertheless important to prevent further vision loss.

This case is consistent with the finding that IOI with brolucizumab can occur after the first or any subsequent injection [15, 20, 24, 25]. It can manifest as anterior, posterior, or panuveitis, ranging from anterior cellular reaction to optic nerve inflammation or to retinal vasculitis [29, 30]. The onset of the AE can range from the day after, to a month or more after injection [15, 20]. Furthermore, the inflammation can occur quite suddenly [14]. We believe it is necessary to manage the patient carefully and aggressively and rule out other infectious and noninfectious causes [7, 13]. For example, in this patient we needed to rule out giant cell arteritis, given her age. Once other etiologies are ruled out, aggressive therapy is needed to manage the inflammation. Both systemic and intravitreal steroids should be considered. Local therapy should also be considered when the time is appropriate. Immunomodulatory therapy may also be indicated. Eye care professionals should consider prompt and intensive treatment of IOI, applying standard-of-care treatment guidelines. In cases of IOI, including retinal vasculitis and/or retinal vascular occlusion, brolucizumab should be discontinued [18]. In addition, it must be remembered that brolucizumab is contraindicated in eyes with active IOI [11, 18].

## Conclusions

A large unmet need still exists for patients with nAMD, with patients experiencing unresolved fluid, high injection burden, and drop-off in adherence [2, 4, 5]. Brolucizumab addresses these unmet needs by providing robust vision gains and superior fluid resolution, with the potential for longer treatment intervals [8, 9]. Through the coalition findings and other work, Novartis has made significant progress in better understanding IOI-related AEs associated with brolucizumab [7, 15–17, 20, 21, 31]. The risks of AEs and vision loss may be mitigated at clear steps along the patient journey. When patients are selected for treatment with brolucizumab, it is important to weigh the potential benefit against risk of AEs and to consider patient risk factors such as prior history of IOI and/or retinal vascular occlusion and female sex [16, 17]. To mitigate the potential for IOI-related AEs following brolucizumab injection, it is important to conduct a thorough dilated eye examination before each injection and closely monitor patients throughout treatment [19, 20]. Brolucizumab should not be injected in the presence of active IOI [11, 18]. It has been shown that most IOI events occur in the first 6 months of treatment [15]. Patients should be educated on which symptoms to monitor for [11, 20]. If an AE is identified, prompt and intensive treatment should be considered [7, 19, 21], and brolucizumab should be discontinued in cases of IOI, including retinal vasculitis and/or retinal vascular occlusion [18].

## Data Availability

Not applicable.

## Abbreviations

AMD: Age-related macular degeneration
BCVA: Best corrected visual acuity
bid: Twice per day
IOI: Intraocular inflammation
MedDRA: *Medical Dictionary for Regulatory Activities*
nAMD: Neovascular age-related macular degeneration
OCT: Optical coherence tomography
OD: Right eye
OS: Left eye
q: Every
qid: Four times per day
SRC: Safety review committee
US: United States
VEGF: Vascular endothelial growth factor
